# Employers' Subscriptions at Sheffield

**Published:** 1920-07-17

**Authors:** 


### Employers' Subscriptions at Sheffield.

MR. C. SIDDALL'S DECLARATION.

THE financial position of the Sheffield Royal Infirmary has caused a useful suggestion to be made which gave unexpected importance to the recent quarterly meeting. The Chairman, Mr. H. H. Bedford, advocated a small charge upon patients as an emergency measure which would not be imposed on poor people, whereupon Mr. C. Siddall suggested an alternative.

He was most anxious, he said, that the voluntary system should be continued, and said that Sheffield people did not subscribe because the hospitals' needs were not explained to them. The small amount of some firms' contributions was explained by the indifference of the Royal Infirmary's officers. He supported this contention by reference to his own case. His own business, he said, had been contributing a miserable six guineas a year; but now that the position had been explained it was subscribing £105 annually. He himself would have known nothing of the necessity had he not been appointed a member of the Board.

Monthly collections, Mr. Siddall added, were hoped for from the workmen who had previously contributed nothing, and by this means £350 a year could be raised.

The business firms in Sheffield are to be circularised; and we hope that Mr. Braime's well-thought-out scheme for an Employers' Contribution Fund, which has proved so successful at Leeds, will be the model of the circular in preparation. It was published in THE HOSPITAL, February 7, 1920, p. 437.

